# Panx3 deficiency in mice impairs bone fracture healing and causes transient hypoglycemia in neonatal animals

**DOI:** 10.1186/s10020-026-01458-9

**Published:** 2026-03-19

**Authors:** Shan Jiang, Anke Baranowsky, Julia Luther, Brooke L. O’Donnell, Brent Wakefield, Clara Bassen, Mona Neven, Gesine Eis-Janzyk, Michael Amling, Silvia Penuela, Thorsten Schinke, Johannes Keller

**Affiliations:** 1 Department of Trauma and Orthopedic Surgery, University Medical Center Hamburg-Eppendorf, Hamburg, 20251 Germany; 2 Department of Osteology and Biomechanics, University Medical Center Hamburg-Eppendorf, Hamburg, 20251 Germany; 3 Department of Anatomy and Cell Biology, Schulich School of Medicine and Dentistry, University of Western Ontario, London, ON Canada

**Keywords:** Fracture Healing, Hypoglycemia, Panx3, C1qtnf3

## Abstract

**Background:**

Panx3, belonging to the pannexin family of channel-forming transmembrane proteins, was previously identified predominantly expressed by bone-forming osteoblasts. Surprisingly however, only neonatal *Panx3*-deficient mice were found to display impaired ossification, whereas the skeleton was unaffected in adult animals. Furthermore, newborn *Panx3*-deficient mice displayed transient hypoglycemia, which was normalized at the age of 4 weeks, suggesting a potential role of Panx3 in linking glucose metabolism to bone growth. As bone regeneration largely recapitulates endochondral ossification during skeletal development, and as both processes require substantial amounts of energy, in the present study we investigated the role of Panx3 in fracture healing using a femoral osteotomy model.

**Methods:**

Female WT and *Panx3*-deficient mice, aged 12 weeks, were subject to a standardized femoral osteotomy. Healing outcomes were measured using µCT and non-decalcified histology, followed by gene expression analysis and genome-wide transcriptomics. In addition, serum samples were collected from patients with fracture or nonunion as well as healthy controls for the measurement of C1QTNF3.

**Results:**

In WT mice, expression of *Panx3* was upregulated in the fractured callus during the course of fracture healing. *Panx3*-deficient mice displayed a striking impairment of bone regeneration, associated with a dysregulated inflammatory response and decreased type-H vessel formation. Transcriptomic analysis of the facture callus further identified differential expression of genes involved in glucose homeostasis, including *C1qtnf3*, which encodes a glucose-lowering adipokine. Although serum glucose levels in *Panx3*-deficient animals were moderately reduced during fracture healing, the most pronounced hypoglycemia was found in neonatal *Panx3*-deficient mice, which also displayed a three-fold increase in serum C1qtnf3 concentrations. Last, we observed increased serum level of C1QTNF3 in patients with fractures compared to healthy controls, and decreased concentrations in patients with nonunion.

**Conclusions:**

Taken together, our data demonstrate that Panx3, at least in mice, is not only essential for skeletal fracture healing, but also involved in the previously established regulation of energy metabolism by osteoblasts.

## Background

Skeletal development, growth, remodeling and regeneration are highly complex processes involving many different cell types with unique physiological functions (Aghajanian and Mohan 2018). Of particular importance in this regard are the bone-forming osteoblasts, which derive from mesenchymal progenitor cells, align at existing bone surfaces and produce the bone extracellular matrix, which subsequently mineralizes (Karsenty et al. 2009). A subset of osteoblasts undergoes terminal differentiation into osteocytes, which form a cellular network within the mineralized bone matrix that regulates bone remodeling and mineral homeostasis (Dallas et al. 2013). The other relevant cell type involved in bone remodeling is the osteoclast, which is generated by fusion of hematopoietic progenitor cells and required to resorb mineralized bone by secretion of hydrochloric acid and matrix-degrading enzymes (Cappariello et al. 2014). The fact that osteoblasts and osteoclasts are entirely different cell types is also relevant for the treatment of osteoporosis, the most prevalent skeletal disorder, which is the consequence of a relative increase of bone resorption over bone formation (Hoong et al. 2025). In fact, osteoporotic patients can be treated in two different ways, namely anti-resorptive or osteoanabolic (Khosla and Hofbauer 2017). Although specific therapeutic options are now established to inhibit bone resorption (bisphosphonates, denosumab) or to activate bone formation (teriparatide, romosozumab), there are still some limitations, especially with respect to osteoanabolic treatment options, which underscores the importance of identifying proteins with specific function in osteoblasts (McClung 2025).

Interestingly, there is a growing body of evidence showing that bone does not only function as a target organ for a broad range of systemic factors, but also acts as an endocrine organ itself (Szeliga et al. 2024). For instance, various studies, in mice and humans, clearly demonstrated that fibroblast growth factor 23 (FGF23) is secreted by osteocytes and functions as the key hormone controlling phosphate homeostasis in the kidney (Agoro and White 2023). Another bone-derived hormone, at least in mice, appears to be osteocalcin, which is produced by osteoblasts, released and activated by osteoclasts and acts on different target organs to control energy metabolism, male fertility, behavior and muscle function (Liu et al. 2016; Determe et al. 2025). Of note, this endocrine function of the skeleton was initially identified by a coincidental observation, i.e. that osteoblast-specific inactivation of the protein tyrosine phosphatase (OST-PTP) results in perinatal lethality due to hypoglycemia (Lee et al. 2007). Subsequent studies then demonstrated that OST-PTP is required to dephosphorylate the insulin receptor in osteoblasts, thereby inhibiting insulin-activated bone resorption and osteocalcin (Ocn) activation (Ferron et al. 2010). Moreover, other bone-derived molecules have also been shown to be involved in regulating energy homeostasis, at least in mice (Suchacki et al. 2020; Mosialou et al. 2017).

On the other hand, it was shown that both bone remodeling and regeneration depend on the provision of large amounts of energy. In fact, several studies clearly demonstrated that osteoblasts are highly glycolytic with a rate of glucose utilization comparable to liver hepatocytes (Zoch et al. 2016). It was additionally reported that osteoblast-mediated bone matrix production is largely dependent on Glut1-mediated glucose uptake, particularly during embryonic development (Wei et al. 2015). Consistent with these findings, osteoblasts do not only express insulin receptors but also respond to osteoanabolic agents with increased glucose consumption (Fulzele et al. 2010). In this regard, it is important to notice that bone tissue, especially during skeletal growth with rapid formation of new bone, accounts for a significant amount of glucose uptake compared to the rest of the organism (Zoch et al. 2016). Taken together, these collective findings have highlighted the existence and potential relevance of a molecular crosstalk between bone cells and glucose homeostasis, which warrants further investigation.

In a screen for osteoblast-specific transmembrane proteins we have previously identified *Panx3*, which we found predominantly expressed in the skeleton, which was not the case for *Panx1* or *Panx2* (Yorgan et al. 2019). To define the physiological functions of Panx3 we generated a mouse deficiency model, in which we only observed a transient phenotype in neonatal animals. More specifically, where there was a clear defect of endochondral ossification observed in *Panx3*-deficient newborn mice, adult animals lacking Panx3 did not display any bone remodeling abnormality. Additionally, about 30% of *Panx3*-deficient mice died in the first two days after birth, which was potentially explained by our observation that serum glucose levels were significantly reduced in newborn animals. In the present study we followed up on our previous analysis and particularly focused on the question if Panx3 deficiency would impact the process of bone regeneration, since we observed that *Panx3* expression was strongly increased during fracture healing.

## Results

### Combined deficiency of Panx1 and Panx3 does not affect bone remodeling in adult mice

**Fig. 1:**
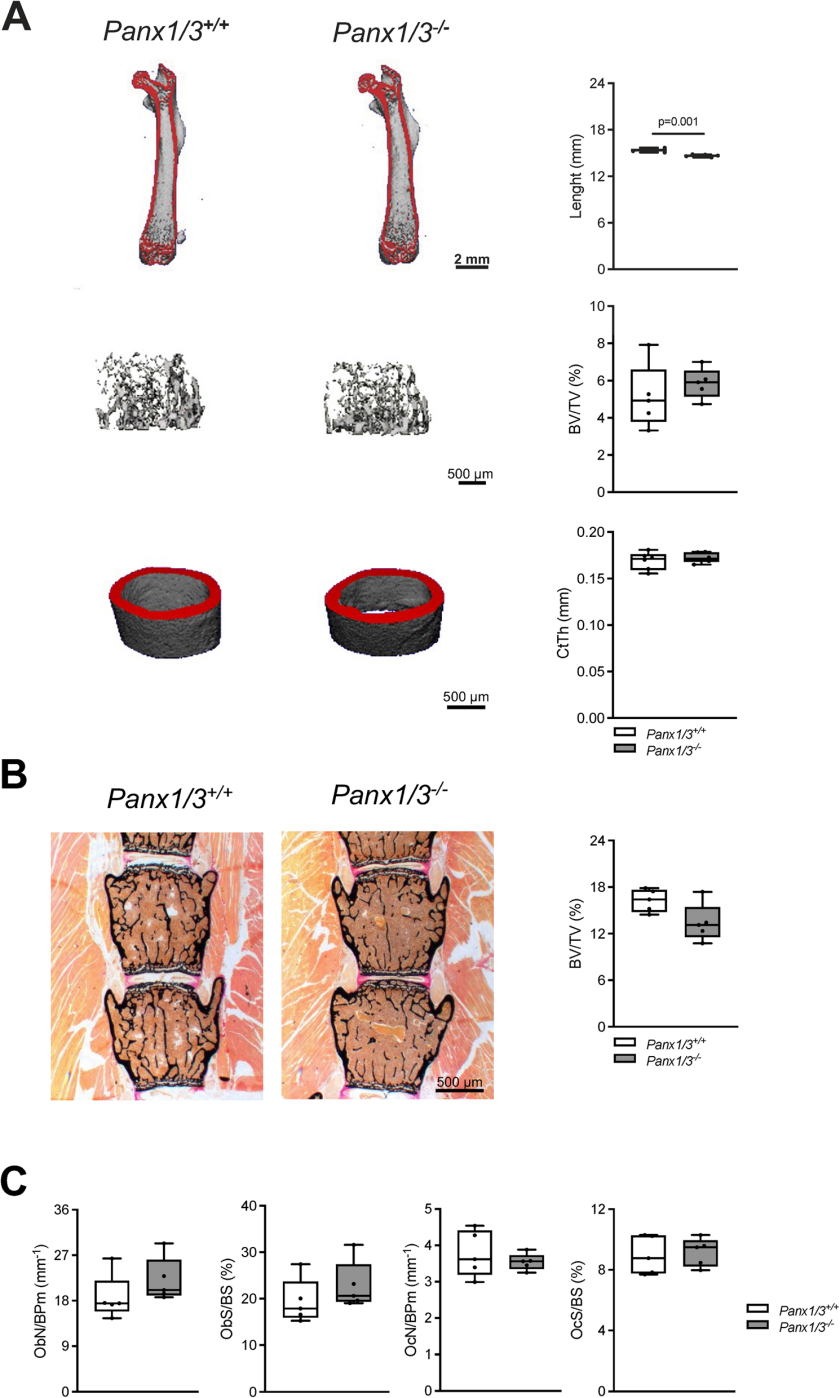
Combined *Panx1/Panx3* deficiency does not affect bone metabolism in adult mice. **A** Representative µCT images of the whole femur (upper row), trabecular bone from the distal femur (middle row) and cortical bone of the femoral midshaft (lower row) from mice of the indicated genotypes at the age of 12 weeks. The length of the whole femur, bone volume per tissue volume (BV/TV) of the trabecular bone and thickness of the cortical bone (CtTh) are presented on the right. **B** Von Kossa/van Gieson staining of undecalcified vertebra sections (L3-4) from the indicated groups and quantification of bone volume fraction (BV/TV) in the same mice. **C** Histomorphometric analysis of osteoblast number per bone perimeter (ObN/BPm), osteoblast surface per bone surface (ObS/BS), osteoclast number per bone perimeter (OcN/BPm) and osteoclast surface per bone surface (OcS/BS) in trabecular bone of spine samples derived from the indicated groups. Unpaired Student’s t test was used to determine statistical differences. For all panels, *N* = 5 per group, data are presented as box plots that represent median with minimum and maximum whiskers

As our previous analysis of gene expression in calvarial bones revealed increased expression of *Panx1* (not of *Panx2*) in *Panx3*-deficient embryos at 19.5 dpc, we first addressed the question if the absence of a bone remodeling phenotype in adult *Panx3*-deficient mice is explained by a compensatory action of Panx1. We therefore analyzed 12-week-old mice lacking both Panx1 and Panx3, for which we had previously observed a moderate impairment of skeletal growth and skull shape (Abitbol et al. 2019). In the present study we applied µCT scanning of the femoral bones and thereby confirmed that *Panx1/3*-deficient mice displayed a reduced femur length (Fig. 1A). Importantly, however, neither the trabecular bone volume per tissue volume (BV/TV) nor the cortical thickness (CtTh.) was significantly different compared to the respective controls. We also analyzed vertebral body sections by undecalcified histology, but we did not observe a difference in the trabecular bone volume either (Fig. 1B). Likewise, there were no differences observed for osteoblast and osteoclast parameters, which were histomorphometrically quantified on the spine sections (Fig. 1C). Taken together, these findings further supported our previous conclusion that Panx3 is dispensable for bone remodeling regulation in adult mice, and the same applies for Panx1.

### Delayed fracture healing in Panx3-deficient mice

**Fig. 2:**
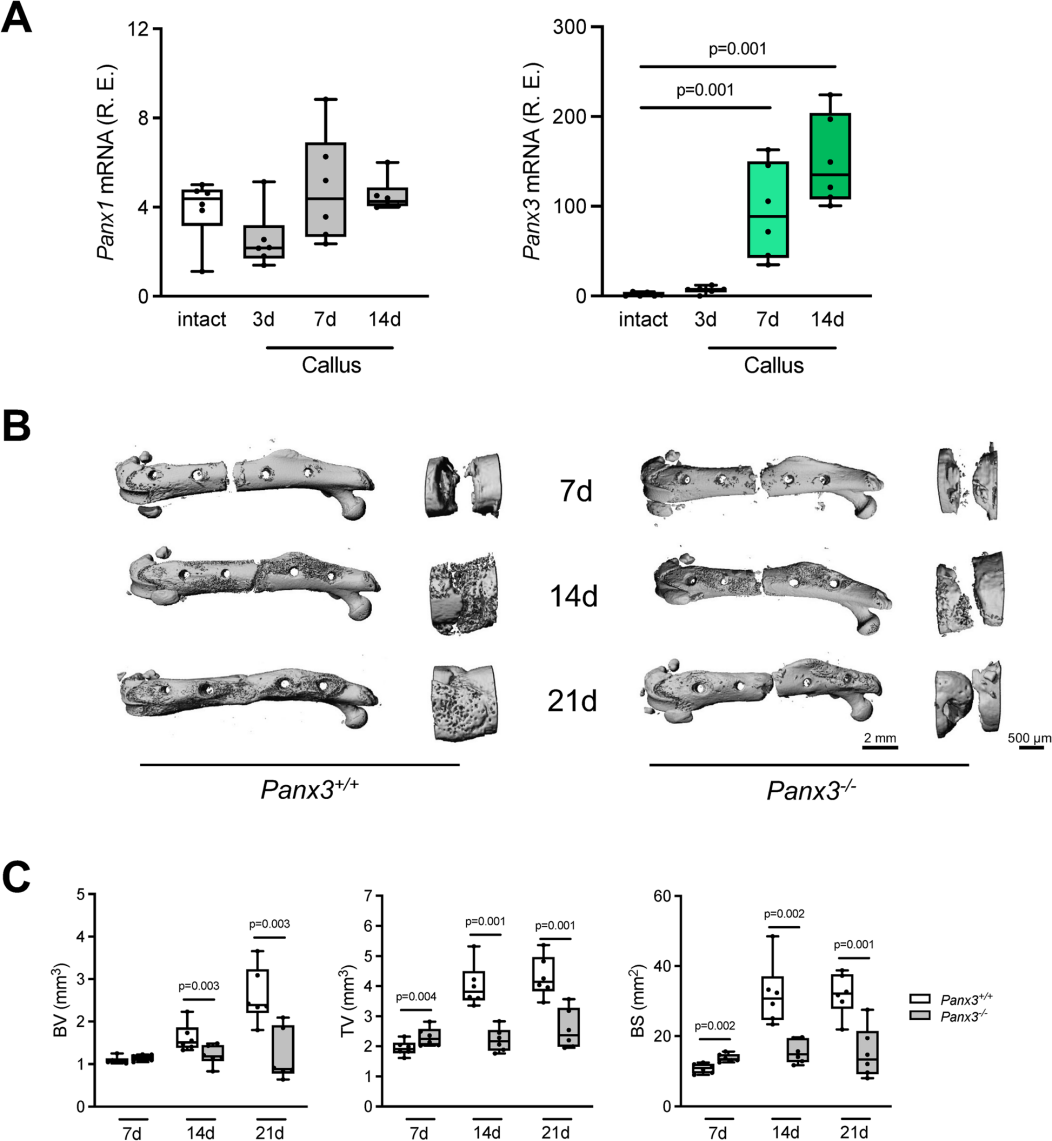
*Panx3* expression is induced in regenerating bone and essential for fracture healing. **A** Gene expression analysis of *Panx1* and *Panx3* in the intact femoral midshaft and the fracture callus, respectively, harvested at the indicated time points postoperatively from WT mice. **B** Representative µCT images of fractured femur and callus of the indicated groups 7, 14 and 21 days after osteotomy. **C** µCT quantification of bone volume (BV), tissue volume (TV) and bone surface (BS) of callus tissue from the indicated groups and time points postoperatively. For **A** ordinary one-way ANOVA and for **C** unpaired two-tailed students t-test for comparison between groups at each time point was used to calculate differences. For **A** and **C** *N* = 6 per group, data are presented as box plots that represent median with minimum and maximum whiskers

**Fig. 3:**
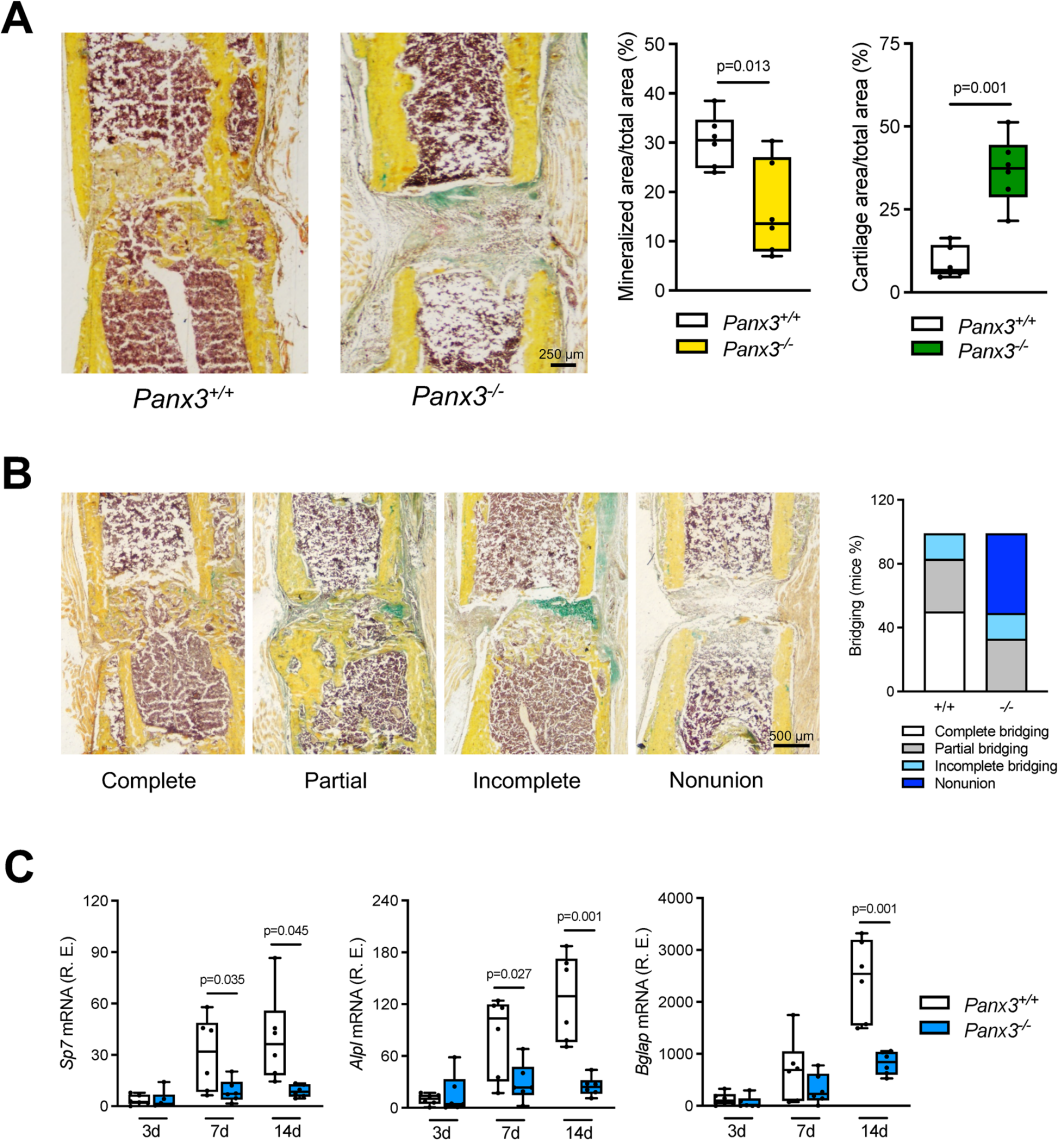
Disturbed callus mineralization and fracture bridging in mice lacking *Panx3*. **A** Representative images of non-decalcified callus sections in the indicated groups stained with Movat Pentachrome (yellow = mineralized bone; green = cartilage; red = muscle and bone marrow), and histomorphometric quantification of mineralized and cartilage tissue in the callus at 21 days postoperatively. **B** Representative images of different fracture healing outcome scenarios and semi-quantitative evaluation of respective osseous bridging status of callus in the indicated groups 21 days after the osteotomy. **C** Relative expression levels of the indicated bone formation markers (*Sp7* = osterix, *Alpl* = alkaline phosphatase, *Bglap* = bone gamma-carboxylglutamic acid-containing protein, osteocalcin) in the callus of *Panx3*^+*/*+^ and *Panx3*^−/−^ mice at 3, 7 and 14 days postoperatively. Unpaired Student’s t test was used to determine statistical differences between groups at each time point. For all panels *N* = 6 per group, data are presented as box plots that represent median with minimum and maximum whiskers

Since skeletal fracture healing essentially recapitulates the process of endochondral ossification, we next addressed the question if deficiency of Panx3 would impair bone regeneration after femur osteotomy. The relevance of this question was further supported by RT-qPCR monitoring gene expression in the fracture callus of wildtype mice collected at different time points after surgery. Here we identified a remarkable induction of *Panx3* expression over time, whereas *Panx1* expression did not substantially change during the course of fracture healing (Fig. 2A). We next applied the femur osteotomy model in 12-week-old wildtype and *Panx3*-deficient mice and monitored the healing process by different methods. As evidenced by µCT-scanning, *Panx3*-deficient mice showed severely impaired mineralization of the fracture gap, which was evident 14 and 21 days after osteotomy (Fig. 2B). Quantification of callus parameters revealed a significantly reduced bone volume (BV), tissue volume (TV), and bone surface (BS) in the callus of *Panx3*-deficient mice (Fig. 2C). These findings were confirmed on histological level, with *Panx3*-deficient mice exhibiting reduced mineralized area per total area in addition to increased cartilage area per total area in the callus (Fig. 3A). Semiquantitative assessment of callus bridging further demonstrated a high rate of fracture nonunion in *Panx3*-deficient mice (Fig. 3B). On molecular level, these morphological alterations were accompanied by a strongly reduced expression of the osteoblast marker genes osterix (*Sp7*), alkaline phosphatase (*Alpl*), and osteocalcin (*Bglap*) at 7 and 14 days after osteotomy (Fig. 3C). Together, these findings demonstrated severely impaired bone regeneration in *Panx3*-deficient mice, characterized by insufficient callus bone formation and mineralization.

### Dysregulation of post-traumatic inflammatory response and type-H vessel formation in the callus of Panx3-deficient mice

**Fig. 4:**
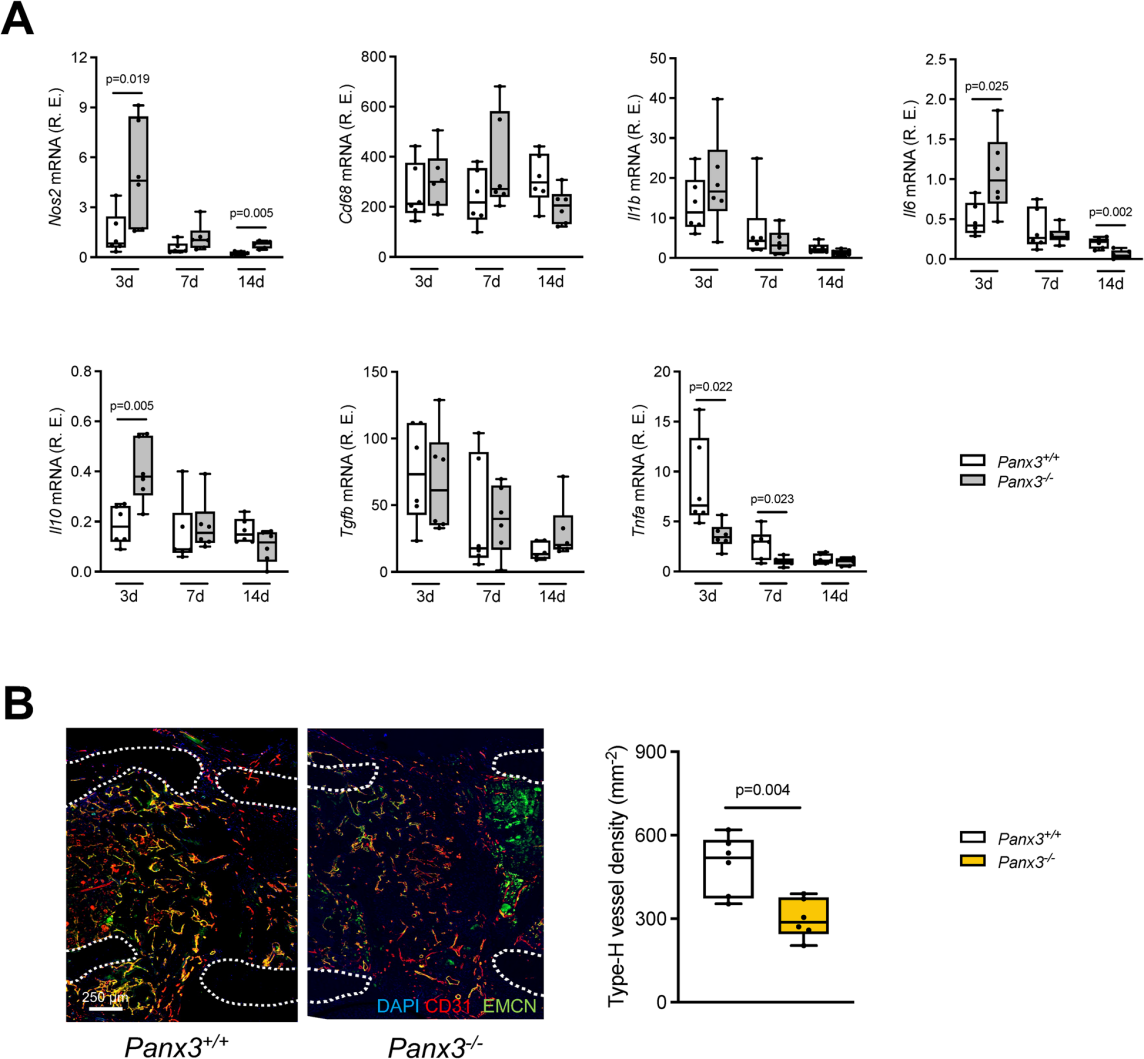
Deficiency of *Panx3* disrupts the post-traumatic immune response and is associated with reduced callus neo-angiogenesis. **A** Expression of inflammation-related genes (*Nos2* = nitric oxide synthase 2, *Cd68* = cluster of differentiation 68, *Il* = interleukin, *Tgfb* = transforming growth factor β, *Tnfa* = tumor necrosis factor α) in the callus of *Panx3*^+*/*+^ and *Panx3*^−/−^ mice at the indicated time points postoperatively. **B** Representative immunohistochemical staining for CD31 and endomucin (EMCN) and quantification of type H vessel density in the fracture callus in mice of the indicated genotypes 14 days postoperatively. Unpaired Student’s t test was used to determine statistical differences between groups at each time point or in each type of tissue. For all panels *N* = 6 per group, data are presented as box plots that represent median with minimum and maximum whiskers

Monitoring the expression of pro- and anti-inflammatory cytokines essential for the post-traumatic inflammatory response in callus, we observed increased expression of pro-inflammatory nitric oxide synthetase 2 (*Nos2*) at day 7 and 21, whereas tumor necrosis factor alpha (*Tnfa*) was decreased 7 and 14 days after fracture in *Panx3*-deficient mice (Fig. 4A). In contrast, interleukin-6 (*Il6*) was increased at day 7 but decreased at day 21 after osteotomy, while anti-inflammatory interleukin-10 (Il10) was expressed at higher levels in mutant animals. These findings were supported by an assessment of callus neo-angiogenesis, where immunofluorescent co-staining of endomucin and CD31 revealed significantly lower density of type-H vessel formation in *Panx3*-deficient mice (Fig. 4C). Together, as adequate fracture healing is thought to depend on metabolic coupling between callus neo-vascularization and osteogenesis (Brakel et al. 2024), the data suggest that this process might be impaired by dysregulated immune response after fracture in a *Panx3*-dependent manner.

### Differential expression of genes involved in glucose homeostasis in the fracture callus of Panx3-deficient mice

**Fig. 5:**
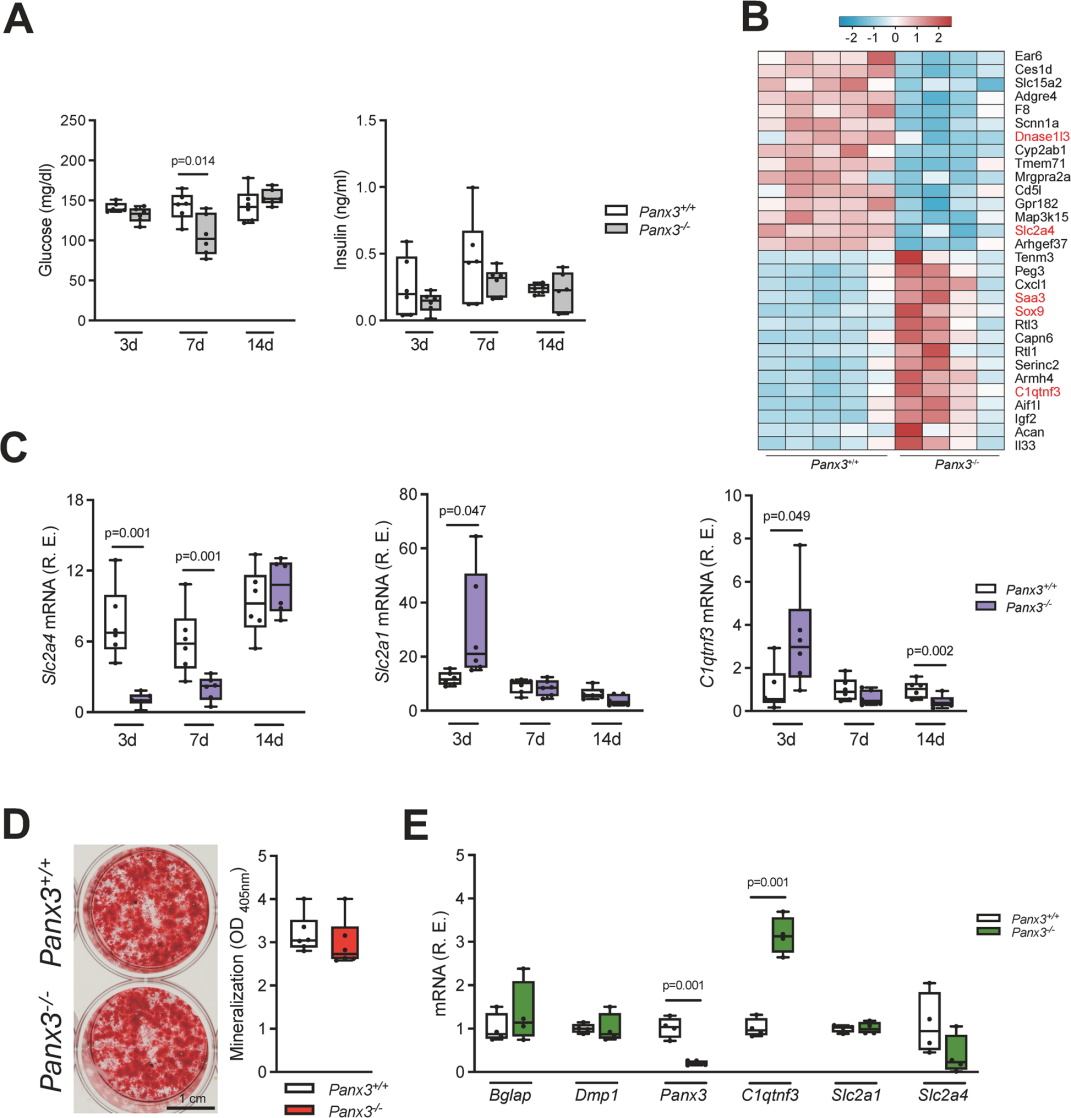
*Panx3* deficiency results in altered glucose metabolism markers.** A** Serum levels of glucose and insulin in mice of the indicated genotypes at 3, 7 and 14 postoperative days (*N* = 6). Relative fold changes to controls were plotted. **B** Heatmap of 30 differentially regulated genes with highest fold changes in the callus of *Panx3*^+/+^ (*N* = 5) and *Panx3*^−/−^ (*N* = 4) mice 3 days after the osteotomy. Each column represents one individual sample and gene, respectively. Z-scores were calculated based on the expression level of each gene and visualized in a color scale (blue = downregulated, red = upregulated). Genes associated with glucose metabolism were highlighted in red. **C** Expression of the indicated genes (*Slc* = solute carrier family, *C1qtnf3* = C1q tumor necrosis factor-related protein 3) in the callus of *Panx3*^+/+^ and *Panx3*^−/−^ mice at the indicated postoperatively time points (*N* = 6). **D** Representative alizarin red–stained images of isolated calvarial osteoblasts from *Panx3*^+*/*+^ and *Panx3*^−/−^ mice and quantification of extracellular matrix mineralization after 10 days of osteogenic differentiation (*N* = 6). **E** Expression of the indicated genes (*Bglap* = bone gamma-carboxylglutamic acid-containing protein, osteocalcin, *Dmp1* = dentin matrix acidic matrix phosphoprotein 1, *Panx3* = pannexin 3, *C1qtnf3* = C1q tumor necrosis factor-related protein 3, *Slc* = solute carrier) in calvarial osteoblasts from *Panx3*^+*/*+^ and *Panx3*^*−/−*^ mice after 10 days of osteogenic differentiation (*N* = 4). Unpaired Student’s t test was used to determine statistical differences between groups at each time point or for each gene. Data are presented as box plots that represent median with minimum and maximum whiskers

Since we have previously observed reduced glycemia in newborn *Panx3*-deficient mice, we also measured serum glucose concentrations in wildtype and Panx3-deficient animals at different stages of fracture healing. Here we observed a significant reduction in *Panx3*-deficient mice glucose levels 7 days after osteotomy, which was not accompanied by altered insulin concentrations in the serum (Fig. 5A). In this context it was additionally interesting that an RNA-sequencing approach identified two potentially relevant transcriptional changes in the fracture callus of *Panx3*-deficient animals (Fig. 5B), which were subsequently confirmed by RT-qPCR (Fig. 5C). More specifically, whereas expression of *Slc2a4*, encoding the glucose transporter GLUT4, was reduced in the callus of *Panx3*-deficient mice, there was an increased expression of C1q/tumour necrosis factor-related protein-3 (*C1qtnf3)*, encoding a glucose-lowering adipokine also known as CRTP3. To analyze these genes for Panx3-dependent regulation, we also isolated primary calvarial osteoblasts from 5-day-old wildtype and *Panx3*-deficient mice and differentiated them for 10 days ex vivo. Here we did not identify a significant impairment of matrix mineralization (Fig. 5D), which was also supported by comparable expression of the osteoblast differentiation markers *Bglap* and *Dmp1* (Fig. 5E). Remarkably, however, whereas *Slc2a1* and *Slc2a4* expression were not significantly different between wildtype and *Panx3-*deficient osteoblasts, *C1qtnf3* expression was markedly increased in the absence of Panx3.

### Impaired glucose homeostasis in Panx3-deficient mice

**Fig. 6:**
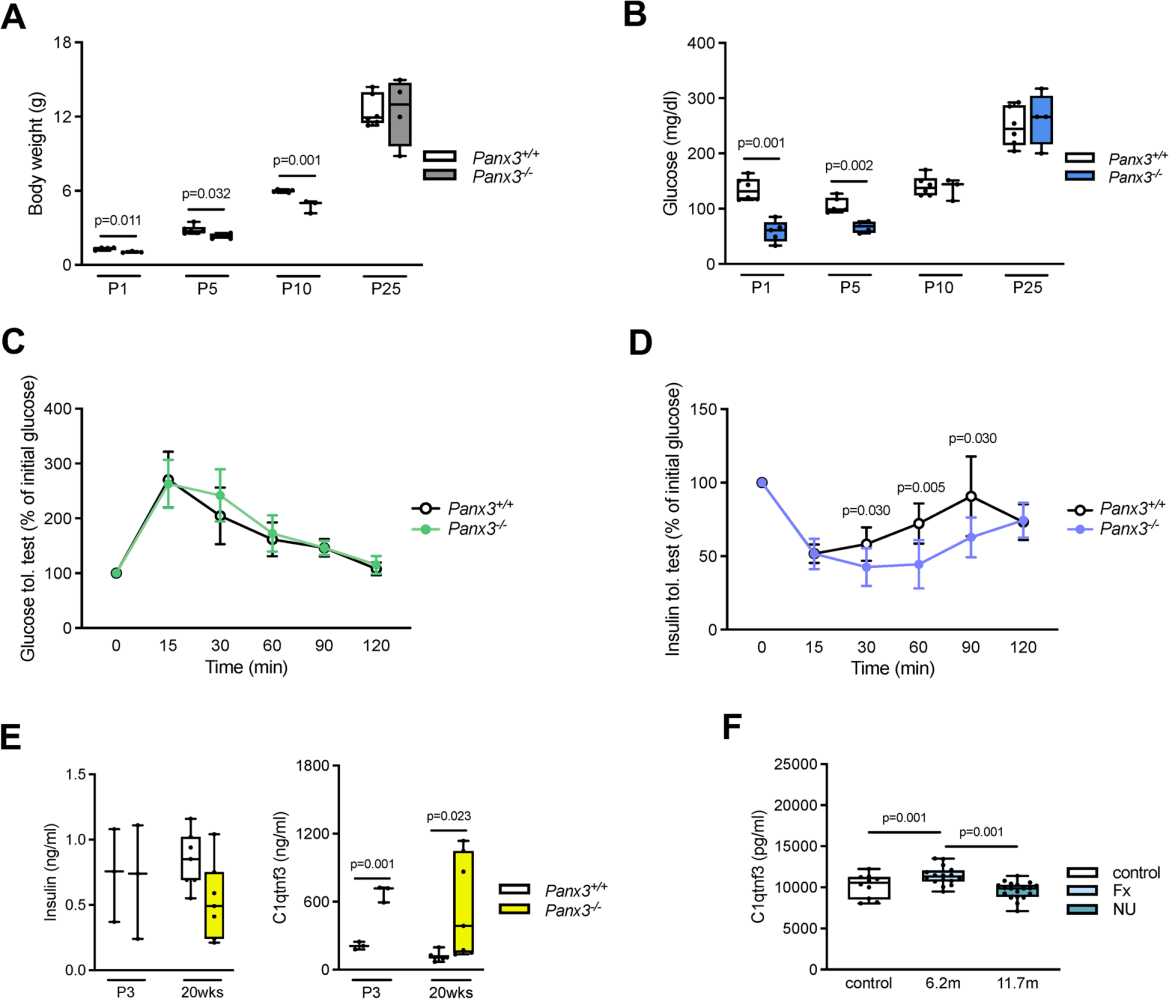
*Panx3*-deficient mice display impaired glucose homeostasis. **A** Body weight of *Panx3*^+*/*+^ (*N* = 4–6) and *Panx3*^−/−^ (*N* = 3–4) mice and **B** serum glucose of *Panx3*^+*/*+^ (*N* = 6) and *Panx3*^−/−^ (*N* = 3–5) mice at the indicated ages. **C** Glucose and **D** insulin tolerance test of adult mice of the indicated genotypes (*N* = 7). Relative alterations to the baseline level were plotted. **E** Serum levels of insulin and C1qtnf3 in *Panx3*^+*/*+^ and *Panx3*^−/−^ mice at the indicated ages (*N* = 3 for P3, *N* = 7 for P600). **F** Serum concentration of C1qtnf3 in healthy individuals as control (*N* = 10), patients displayed adequate fracture healing (Fx, *N* = 16) and nonunion formation (NU, *N* = 19). Unpaired Student’s t test was used to determine statistical differences between groups at each time point. Data are presented as box plots that represent median with minimum and maximum whiskers

This unexpected finding led us to focus more specifically on the early postnatal stages, since our previous study was mostly concentrating on the absence of a bone remodeling phenotype in adult *Panx3*-deficient mice (Yorgan et al. 2019). We analyzed wildtype and *Panx3*-deficient littermates 1, 5, 10 or 25 days after birth. Consistent with the previously reported delayed ossification phenotype, there was a significantly reduced body weight in *Panx3*-deficient animals until the age of 10 days, whereas there was no difference between wildtype and *Panx3*-deficient mice 25 days after birth (Fig. 6A). Most importantly, there was a remarkable reduction of serum glucose levels in *Panx3*-deficient neonates, but there was no significant difference towards wildtype littermates at day 10 and 25 after birth (Fig. 6B). To analyze for potential impairment of glucose homeostasis in adult *Panx3*-deficient mice, we additionally performed glucose and insulin tolerance tests in 18-week-old wildtype and *Panx3*-deficient littermates. Although we did not observe a striking difference between the two groups, there was a significantly delayed normalization of serum glucose after insulin injection in adult *Panx3*-deficient mice (Fig. 6C,D).

### Altered C1qtnf3 serum concentrations in Panx3-deficient mice and in patients with fracture nonunion

To connect our molecular findings with the impaired glucose homeostasis phenotype of *Panx3-*deficient mice, we measured insulin and C1qtnf3 concentrations in the serum of newborn (postnatal day 3) and adult (20 weeks old) wildtype and *Panx3*-deficient littermates. At both ages, we found that insulin concentrations were not different between the two groups (Fig. 6E). Importantly, however, there was a remarkable increase of C1qtnf3 serum concentrations in 3-day-old *Panx3*-deficient animals, whereas this was more variable in the adult group. Finally, since *C1qtnf3*, similar to *Panx3*, was previously found to be one of the most strongly induced genes during fracture healing of mouse bones (Coates et al. 2019), we took advantage of an existing set of serum samples from patients with healing and non-healing skeletal fractures. For patients with healing fractures, serum samples were taken at the final clinical follow-up, which occurred on average 6.2 months after the fracture. For patients with non-healing fractures, serum samples were taken before revision surgery at a mean time of 11.7 months after the initial fracture. Despite the time gap between the two time points, C1QTNF3 serum concentrations were increased in patients with healed fractures compared to controls, whereas C1QTNF3 serum levels were unaltered in patients with inadequate healing.

## Discussion

We have previously identified Panx3 in a screening approach for genes with predominant expression in bone-forming osteoblasts and subsequently identified a remarkable bone-specific expression pattern in adult wildtype mice (Yorgan et al. 2019). Since this finding essentially predicted a physiological role of Panx3 in the regulation of bone formation, we generated a *Panx3*-deficient mouse model, in which we did not observe any bone remodeling pathology at the ages of 4 or 24 weeks. Importantly, however, we identified delayed endochondral ossification in newborn *Panx3*-deficient animals, together with a lethality rate of nearly 50% in the first two days after birth (Yorgan et al. 2019). Since osteoblasts have previously been reported to regulate glucose homeostasis, we additionally measured serum glucose levels in newborn mice, thereby identifying hypoglycemia in *Panx3*-deficient neonates, which was not observed at the age of 4 weeks. Although it is unclear if this transient pathology explains the high lethality rate of newborn *Panx3*-deficient mice, our findings were consistent with other publications, in which not only high expression of *Panx3* in skeletal types was reported, but also delayed ossification of *Panx3*-deficient mice as well as early postnatal lethality in a subset of animals (Abitbol et al. 2019; Ishikawa et al. 2016; Moon et al. 2015).

In the present study we followed up on our previous findings and eventually addressed three main questions. First, since we have previously found that *Panx1* expression is increased in *Panx3*-deficient embryos at 19.5 dpc, we analyzed animals lacking both *Panx* genes for bone remodeling disturbances at 12 weeks of age. Second, since skeletal fracture healing essentially recapitulates the processes required for endochondral ossification, we analyzed the possibility that bone regeneration is impaired in 12-week-old *Panx3*-deficient mice. Third, also triggered by the RNA sequencing results of the fracture callus, we expanded our analysis on the glucose metabolism phenotype of newborn *Panx3*-deficient mice and also identified increased C1qtnf3 production as a possible molecular cause. Although there are still several issues that remain to be addressed by future experiments, it is evident that i) Panx3 is not required for bone remodeling regulation in adult mice, ii) Panx3 deficiency causes a profound negative effect on skeletal fracture healing, and iii) hypoglycemia is a major, albeit transient phenotype in newborn *Panx3*-deficient mice.

With respect to the *Panx1/3*-deficient mouse model we have previously reported that the simultaneous deletion of both *Panx* genes moderately affects specific skull shape parameters and reduces the length of long bones (Abitbol et al. 2019). For the present study we focused on trabecular and cortical bone mass parameters by µCT scanning of the femora and on histomorphometric quantification of bone remodeling cell types in undecalcified spine sections. We thereby confirmed that the combined deficiency of Panx1 and Panx3 results in shortened femoral length, but trabecular bone volume, cortical thickness as well as osteoblast and osteoclast indices were not different from wildtype controls. Therefore, this phenotype is essentially identical to the one reported for *Panx3*-deficient mice, which confirms our previous conclusion, that bone remodeling regulation in adult mice does not depend on the presence of Panx3 (Yorgan et al. 2019). On the other hand, as reported previously, the rate of early postnatal lethality was similar in *Panx3*-deficient and in *Panx1/3*-deficient mice, which essentially shows that a compensatory induction of *Panx1* in the absence of Panx3 does not prevent this neonatal pathology. Therefore, we focused our further analyses on the *Panx3*-deficient mouse model, also because only *Panx3*, not *Panx1*, displayed increased expression in the fracture callus of wildtype mice.

Using our standardized femur osteotomy model (Jahn et al. 2024), we observed that Panx3 deficiency caused a profound negative influence on the healing process. More specifically, there was a delayed ossification associated with enrichment of cartilaginous structures in the fracture callus of *Panx3*-deficient mice. Likewise, 50% of the analyzed *Panx3*-deficient animals, displayed fracture nonunion, which is also of potential clinical relevance since causes and/or risk factors for insufficient healing in humans are still poorly understood (Stewart 2019). Collectively, these findings are principally consistent with the delayed ossification phenotype of newborn *Panx3*-deficient animals, thereby suggesting that lack of Panx3 expression is specifically causing pathologies in states of excessive osteoblastogenesis and bone formation. Also remarkable in terms of comparability to newborn stages was the finding that serum glucose levels were significantly lower in *Panx3*-deficient mice at day 7 of fracture healing.

Due to the global Panx3-deficiency model, we are unable to differentiate between local and systemic implications of Panx3-deficieny on bone regeneration, that include potential impairment of local glucose availability in the callus versus a moderate systemic hypoglycemia observed at day 7 after osteotomy in mutant mice. Panx3-deficiency was not only accompanied by severely impaired but also local immune dysregulation and impaired formation of osteogenic type-H vessels. In this regard, it is important to state that both phenomena can either elicit impaired healing, but may also be secondary to impaired callus ossification per se.

Together with the differential expression of *Slc2a4* and *C1qtnf3* in the fracture callus of wildtype and *Panx3*-deficient mice, however, these findings further led us to reassess the hypoglycemia phenotype associated with Panx3 deficiency, since our previous study only included groups of newborn or 4-week-old animals (Yorgan et al. 2019). In the present study we analyzed wildtype and *Panx3*-deficient littermates at four time points after birth. Whereas *Panx3*-deficient animals had reduced body weight until day 10 after birth, they appeared phenotypically normal at weaning age and thereafter. Most importantly, there was a dramatic reduction of serum glucose concentration in newborn and 5-day-old *Panx3*-deficient mice, but this pathology was normalized 10 or 25 days after birth. Taken together, these data fully confirm that transient hypoglycemia is a major phenotype of *Panx3*-deficient mice, which potentially explains their early postnatal lethality. The other remarkable finding in this regard was that serum insulin levels were not different between 3-day-old wildtype and *Panx3*-deficient littermates, but C1qtnf3 concentrations were three-fold higher in animals lacking Panx3. Moreover, despite higher variation, serum levels of C1qtnf3 were also significantly increased in adult *Panx3*-deficient mice. Although it remains to be proven that excessive C1qtnf3 production by *Panx3*-deficient osteoblasts causes the reduction of serum glucose concentrations, these findings are potentially interesting, also in the context of fracture healing, since we identified reduced C1qtnf3 serum concentration in patients with fracture nonunion.

An impact of C1qtnf3, also known as CTRP3, on glucose homeostasis has been shown in various studies (Yaribeygi et al. 2019). First, C1qtnf3 has been reported to reduce blood glucose levels following its administration to wildtype mice (Peterson et al. 2010). Likewise, C1qtnf3 over-expression in transgenic mice protected them from hepatic steatosis when fed a high-fat diet (Peterson et al. 2013). Although the human relevance of these influences are still controversially discussed, there are numerous additional studies for C1qtnf3 as a major regulator of glucose homeostasis, which have been attributed to specific influences on liver and adipose tissues (Guo et al. 2020). With respect to fracture healing it is noteworthy that a transcriptomic analysis identified *Panx3* and *C1qtnf3* as the two most strongly induced genes at day 3, 5 and 7 of the healing process in a murine stress fracture model (Coates et al. 2019). Moreover, based on clinical findings, which suggested a positive correlation of C1qtnf3 with bone mineral density, *C1qtnf3*-deficient mice have already been analyzed for a potential impact on fracture healing in a system using stabilization with an intramedullary pin (Demirtas et al. 2020; Youngstrom et al. 2020). Although not directly comparable to our model using an external fixator, it was found that C1qtnf3 deficiency significantly impairs fracture healing. Since we essentially observed a similar healing pathology (i.e. nonunion) in *Panx3*-deficient mice, in which we identified increased *C1qtnf3* expression, these findings are essentially counter-intuitive, unless they are regarded as a compensatory mechanism to counteract the disturbed healing process. On the other hand, the reduced C1qtnf3 serum concentrations in individuals with nonunion essentially support the findings obtained in *C1qtnf3*-deficient mice, which could be even more relevant from a clinical perspective.

On the other hand, based on the findings reported in the present manuscript, C1qtnf3 can only be regarded as a candidate mediator linking Panx3 deficiency to glucose homeostasis and impaired fracture healing. In fact, it needs to be clearly stated that our study has several limitations and that many additional studies are required to substantiate the relevance of C1qtnf3. First, it remains to be established, which cell types produce C1qtnf3 in *Panx3*-deficient animals, either postnatally or within the fracture callus. This is particularly important, since *C1qtnf*3 is not only expressed in various tissues, but also in chondrocytes (Maeda et al. 2006). Second, our focus on mice with global inactivation of Panx3 does not allow to draw conclusions in this regard either. Therefore, future studies will have to focus on mouse models with conditional inactivation of Panx3, either in osteoblasts or in chondrocytes. Third, we were unable to demonstrate that the increased serum levels of C1qtnf3 are indeed causative for the postnatal hypoglycemia of *Panx3*-deficient mice. Here, it would be most informative to address the question, if additional deficiency of C1qtnf3 would correct the early postnatal phenotype of *Panx3*-deficient mice.

## Conclusion

In conclusion, whereas Panx3 deficiency is not required for postnatal bone remodeling regulation, it negatively affects skeletal fracture healing and early postnatal glucose homeostasis. Although the underlying molecular pathways remain to be identified, we observed a remarkable overproduction of C1qtnf3, especially in neonatal *Panx3-*deficient animals. Whether an increased expression of *C1qtnf3* in *Panx3-*deficient osteoblasts is the main course of the transiently observed hypoglycemia remains to be established in future experiments, ideally by generating mice with a combined deficiency of Panx3 and C1qtnf3.

## Materials and methods

### Animals

*Panx3*^*−/−*^ mice with a pure C57BL/6 J background were generated and genotyped as previously described (Yorgan et al. 2019). Wildtype littermates were employed as control for the comparison of fracture healing outcome. *Panx1/3*^*−/−*^* mice* with a C57BL/6N background were generated and genotyped as previously described (Abitbol et al. 2019), and the skeleton phenotype was compared to a separate set of wildtype mice with the same background. All animals were housed in a specific pathogen-free animal facility with standard conditions at a 12-h light/dark cycle and fed ad libitum. For assessment of skeletal status, mice without surgical interventions were used. For fracture healing studies, mice were assayed for radiologic indices of bone regeneration (µCT scanning; primary endpoint), static and cellular histomorphometric alterations (secondary endpoint), and type-H vessel formation. For all animal experiments, directly compared groups were performed in parallel throughout the experiment. Mice were randomized to the different experimental groups, and all biological samples were coded to facilitate blinded evaluation. Health status was controlled daily, using predefined score sheets and humane end points, whereby no animal had to be excluded. All relevant ethical regulations for animal experiments were complied. All animal experiments were conducted in compliance with the ARRIVE guidelines and with approval from the “Behörde für Justiz und Verbraucherschutz der Freien und Hansestadt Hamburg” (N123/2019, N010/2020).

### Skeletal analysis

For analysis of the intact (i.e., non-fractured) skeleton, female 12-week-old mice without prior surgical intervention were employed. Bone samples were fixed in 3.7% PBS-buffered formaldehyde overnight, washed and transferred in 80% ethanol. For bone histology, the lumbar vertebral bodies L3 to L6 were dehydrated with ascending alcohol concentrations and embedded in methylmethacrylate. Sections of 4 µm thickness in a sagittal plane were cut and stained with von Kossa/van Gieson for static histomorphometry, toluidine blue and TRAP staining for quantification cellular parameters (Jahn et al. 2024). Brightfield images were acquired using Pannoramic MIDI II slide scanner (Sysmex corporation, Hyogo, Japan). BoneJ plugin of ImageJ and Osteomeasure were used for the static and cellular histomorphometry (Domander et al. 2021; Schneider et al. 2012). After osteotomy, fractured femora were fixed 4% paraformaldehyde for 24 h, washed before being infiltrated with an ascending sucrose gradient (10, 20, and 30% each for 24 h at 4 °C) and embedded in super cryo-embedding medium (Section-Lab Co. Ltd.). Cryosections in 5 µm thickness were prepared and stained with Movat Pentachrome. Histomorphometric analysis evaluating the composition of callus were carried out using ImageJ. Semi-quantitative scoring of the callus bridging status was performed as previously described (Appelt et al. 2021). In brief, scoring was performed independently by two blinded reviewers and averages are depicted as stacked bar charts. Complete bridging = all four cortices bridged by callus; partial bridging = two to three cortices bridged by callus; incomplete bridging = callus present, but no bridging visible; nonunion = rounded cortices, minimal presence of callus.

### Surgical procedure

For the fracture model, female mice at the age of 12–14 weeks were subjected to a femoral osteotomy as previously described (Jiang et al. 2021). Briefly, following anesthesia with isoflurane and exposure of the right femur, the midshaft region was determined based on anatomical landmarks, including the greater trochanter and the medial condyle. The positions of the pin holes were carefully measured to ensure alignment of the fixator with the longitudinal axis of the femur and correct centering at the planned midshaft region. Pin holes were then drilled, and the fixator was mounted to the femur. A standardized gap on the femoral midshaft was created using a 0.7 mm-diameter gigli wire saw, followed by flushing the fracture gap and wound closure with a simple interrupted suture. Buprenorphine (0.1 mg/kg) and clindamycin (150 mg/kg) were administered pre-operatively for provision of analgesia and prevention of infection. After surgery, mice were placed in a recovery rack overnight and received drinking water supplemented with 1 mg/ml metamizole for 3 days. Mice were sacrificed at the indicated time points and the fractured femora were harvested for further analysis.

### Micro-CT

After fixation, micro-CT (µCT) scanning was carried out using Scanco vivaCT 80 (SCANCO Medical, Brüttisellen, Switzerland) with a voxel size of 15.6 µm at 70kVp, 113µA and 400 ms integration time. µCT Ray V4.0–4 (Scanco Medical AG, Brüttisellen, Switzerland) were used for the reconstruction of the 3-dimentional overviews and generation of representative images. For trabecular and cortical bone evaluation, the volume-of-interest (VOI) was defined using automatic scripts and calculated using the μCT Evaluation Program V6.6 (Scanco Medical AG, Brüttisellen, Switzerland). For evaluation of the callus, the fracture ends of the cortical bone were used as landmarks, and the center of the fracture gap was determined. The callus was defined as calcified tissue within a 1-mm region (corresponding to 64 slices at a voxel size of 15.6 µm) around the center of the gap. The volume of interest for the callus evaluation was contoured manually on each slide. For evaluation of unfractured femora, a 1-to 2-mm distal and 1-to 2-mm distal midshaft segment were analyzed. Data were reported in compliance with the guidelines for tissue imaging by the American Society of Bone and Mineral Research (Dempster et al. 2013).

### Immunofluorescent staining

For visualization of osteogenic type-H vessels in the fractured callus, immunofluorescent staining of CD31 and Endomucin were performed on cryosections. Briefly, the sections were air-dried for 1 h and washed with PBS, followed by blocking with 3% bovine serum albumin (BSA) and 5% normal donkey serum (#END9000-100, Histoprime) in PBS. The primary antibodies anti-CD31-PECAM (1:200, #AF3628, R&D Systems, Minneapolis, USA) and Endomucin (1:100, #sc-65495, Santa Cruz Biotechnology, California, USA) were incubated overnight at 4 °C. After washing, the sections were incubated with secondary antibodies donkey-anti-goat (1:400, #A11058, ThermoFisher, Massachusetts, USA) and donkey-anti-rat (1:400, #A21208, ThermoFisher, Massachusetts, USA) for 1 h at room temperature. Nuclei staining was performed using Hoechst 33,258 (1:10,000, #H3569, Thermo Fisher Scientific Inc., MA, USA). Sections were stored at 4 °C in darkness until imaging. Immunofluorescent sections were scanned using Sysmex Pannoramic MIDI II (Sysmex Cooperation, Hyogo, Japan) and Sysmex Pannoramic Scanner v3.0.6 software. Sections were imaged using the Olympus IX-83 Evident spinning disc microscope (Evident Scientific Inc., Hamburg, Germany) and Leica TCS SP8 X (Leica Microsystems, Wetzlar, Germany). Images were further processed using ImageJ.

### Cell culture experiments

Primary osteoblasts were isolated by sequential collagenase type 1 A (#C9891, Sigma-Aldrich, Taufkirchen, Germany) and dispase (Dispase Grade ll, #04.942078.001, Roche, Mannheim, Germany) digestion (10 min for each step) of calvariae from 3–5 day-old mice. Whereas cells of the first two digestion steps were discarded, cells from digestion steps 3 to 5 were cultivated in αMEM (#M0644, Sigma-Aldrich, Merck, Darmstadt, Germany) supplemented with 10% FCS and 100U/ml penicillin/streptomycin (#15,140,122, Life Technologies, Carlsbad, USA) (Baranowsky et al. 2022). The cultured cells were differentiated in the presence of 25 µg/mL ascorbic acid and 5 mM β-glycerophosphate. After 10 days of osteogenic differentiation, cells were assayed for mineralization (Alizarin red staining) or gene expression analysis using real-time reverse transcription quantitative polymerase chain reaction (RT-qPCR). Assessment of alizarin red staining was performed as described previously (Baranowsky et al. 2021). Quantification of mineralization was performed using ImageJ.

### Gene expression analysis

For RNA isolation from regenerating bone, the callus was carefully dissected from the femoral midshaft after removal of the surrounding soft tissue, followed by homogenization in TRIzol (#T9424, Sigma Aldrich, Merck, Darmstadt, Germany) using an Ultra Turrax (IKA Labortechnik, Staufen, Germany). Following extraction of total RNA using phenol–chloroform (#P1944, Sigma Aldrich, Merck, Darmstadt, Germany), samples were further purified using the NucleoSpin RNA Kit (#740,955, Macherey–Nagel, Düren, Germany) according to the manufacturer's instructions. Regarding in vitro experiments, total RNA was isolated using the same kit following manufacturer's instructions. RNA concentration and quality were determined using a Nanodrop 2000 spectrophotometer (Nanodrop Technology, Wilmington, MA, USA). Complementary DNA (cDNA) was prepared using the ProtoScript First Strand cDNA Synthesis Kit (#E6300L, New England BioLabs Inc., Ipswich, MA, USA). Quantitative polymerase chain reaction (RT-qPCR) was performed using primers designed for SYBR Select Master Mix for CFX (#4,472,942, Applied Biosystems, Thermo Fisher Scientific Inc., Waltham, USA) or pre-designed probes with the TaqMan Gene Expression Master Mix (#4,369,016, Applied Biosystems, Thermo Fisher Scientific Inc., Waltham, USA). Glyceraldehyde-3-phosphate dehydrogenase (*Gapdh*) was used for the normalization of target genes. Relative expression was presented in arbitrary units (A.U.). The sequences of primers and are listed in (Table 1).

**Table 1:** List of primers used in gene expression analysis

Primers used with SYBR Green	
**Gene**	**sequence**
*Gapdh*	5’-TGACCCCTTCATTGACCTCAAC-3’ 5’-GCATCGCCCCACTTGATTTTG-3’
*Panx1*	5’- CCACCGAGCCCAAGTTCAA-3’ 5’- GGAGAAGCAGCTTATCTGGGT-3’
*Panx3*	5’- GTGATGAGACCCATGTCCCC-3’ 5’- TTTGTCCCACCGACATAGCC-3’
*Sp7*	5’- GCCCCCTGGTGTTCTTCATT-3’ 5’-CCCATTGGACTTCCCCCTTC-3’
*Alpl*	5’-CCAACTCTTTTGTGCCAGAGA-3’ 5’-GGCTACATTGGTGTTGAGCTTTT-3’
*Bglap*	5’-CCTGGCTGCGCTCTGTCT-3’ 5’-TGCTTGGACATGAAGGCTTTG-3’
*Cd68*	5’-TGTCTGATCTTGCTAGGACCG-3’ 5’-GAGAGTAACGGCCTTTTTGTGA-3’
*Il1b*	5’-AGGAGAACCAAGCAACGACAA-3’ 5’-CTTGGGATCCACACTCTCCAGC-3’
*Il6*	5’-GCCTTCTTGGGACTGATGCT-3’ 5’-GAATTGCCATTGCACAACTCTT-3’
*Il10*	5’-AGGCGCTGTCATCGATTTCT-3’ 5’-ATGGCCTTGTAGACACCTTGG-3’
*Tgfb*	5’- CGTCAGACATTCGGGAAGCA-3’ 5’- GCCCTGTATTCCGTCTCCTT-3’
*Tnfa*	5’- GTCCGGGCAGGTCTACTTTG-3’ 5’- AGAGTTGGACCCTGAGCCAT-3’
**Taqman assays**	
**Gene**	**Assay ID**
*Gapdh*	Mm99999915_g1
*Nos2*	Mm00440502_m1
*Slc2a4*	Mm00436615_m1
*Slc2a1*	Mm00441480_m1
*C1qtnf3*	Mm00473047_m1
*Dmp1*	Mm01208363_m1

### RNA-sequencing

Total RNA samples from *Panx3*^+*/*+^ and *Panx3*^*−/−*^ callus at 3 days post-operatively were sent to BGI Genomics for mRNA sequencing. In brief, the transcriptome library was generated according to a standard mRNA library construction procedure with sequencing carried out on the DNBSEQ G400 high-throughput platform with default settings. Quality control and data cleaning were performed using SOAPnuke (v2.3) with commonly adopted filtering criteria. Reads containing adaptor sequences or with an N content greater than 1% were removed, and reads containing more than 40% low-quality bases (Phred score < 20) were filtered out. Clean reads passing these criteria were used for downstream analyses. Subsequently, the filtered clean data was aligned to the reference genome (GRCm39) using Hierarchical Indexing for Spliced Alignment of Transcripts HISAT2 (v2.0.4) with stringent parameters. The sensitive mode was applied, and only uniquely mapped, properly paired reads were retained. The maximum allowed fragment length was set to 1000 bp to reflect the expected insert size distribution and to exclude unlikely long-distance pairings. The statistics of the mapping rate and the distribution of reads on the reference sequence were evaluated to validate the quality of alignment. For gene expression analysis, clean reads were mapped to reference transcripts using Bowtie2 (ver 2.2.5), and expression levels were calculated using RSEM (v1.2.8). Volcano plot, Cluster heatmap and differentially expressed gene (DEG) analysis were carried out using Dr. Tom system and Galaxy web platform (Community 2024). In order to enable a clear and concise analysis of differentially regulated genes that could have a significant biological impact, only the 30 DEGs with the highest absolute fold changes were selected for further evaluation.

### Serum analysis

Whole blood from mice was collected during sacrifice and, after centrifugation, the obtained serum was frozen at −80ºC for further analysis. Glucose levels were determined using the Accu-Check system (Roche Diabetes Care Deutschland GmbH, Germany). Serum concentrations of insulin were determined by ELISA (#KAQ1251, Thermo Fisher Scientific, Waltham, USA). Murine C1qtnf3 levels were measured by using an ELISA kit (#LS-F10970, LifeSpan Biosciences, Newark, USA). C1qTNF3 duo set ELISA (#DY7925-05, R&D Systems Inc., Minneapolis, USA) was used to determine the concentration in human patients. All kits were performed according to manufacturer’s instructions. Tolerance tests for glucose and insulin were performed by oral gavage of glucose solution (1 g/kg bodyweight) or intraperitoneal injection of insulin (0.5U/kg bodyweight), followed by multiple measurements of blood glucose within a 2-h period. All mice were fasted 6 h before glucose and insulin tolerance test.

### Clinical study cohorts and human sample collection

Clinical data and human serum samples were obtained from an ongoing prospective study including patients with nonunion (nonunion cohort) and patients with adequate fracture healing (fracture cohort). Patients undergoing closing-wedge surgery to correct malalignment of the knee joint without other diseases were enrolled as controls (control cohort). Detailed clinical data were collected from all cohorts, including sex, age, body mass index (BMI), previous medical conditions, current medications, smoking habits and previous drug use.

Nonunion cohort: Included were patients (aged 20–84 years) that had received initial treatment of a long bone fracture (clavicula, humerus, radius, ulna, femur, tibia) in external hospitals or in our department and presented for surgical revision of a nonunion fracture at a median of 11.7 months after their injury. Nonunion was diagnosed in the absence of radiographic signs of healing (radiograph and/or CT) and persistent clinical complaints (pain) at least 6 months after surgery. Exclusion criteria were age < 18 years, stress fractures, acute infection, diabetes mellitus type 2, metabolic syndrome, and genetic diseases affecting bone remodeling. Serum samples were taken 1–3 days pre-operatively, centrifuged, aliquoted and frozen at −80 °C. Fracture cohort: patients (aged 20–68) were included who underwent surgical treatment of a long bone fracture at our department and presented for follow-up at a mean time of 6.2 months after surgery. Exclusion criteria were age < 18 or > 80 years, open fractures, stress fractures, concomitant fractures of the axial skeleton, history of nonunion, immunosuppressants, acute infection, diabetes mellitus type 2, metabolic syndrome, or genetic diseases affecting bone remodeling. Serum samples were taken 1–3 days preoperatively, centrifuged, aliquoted and frozen at −80 °C.

Control cohort: samples were collected as controls from otherwise healthy patients (aged 19–65) undergoing closing-wedge surgery to correct malalignment of the knee joint. Exclusion criteria were age < 18 years, any type of disease or drug intake affecting bone, diabetes mellitus type 2, metabolic syndrome, and musculoskeletal injury in the past 3 months. Serum samples were taken 1–3 days preoperatively, centrifuged, aliquoted and frozen at −80 °C.

Informed consent was obtained from every patient. Sample collection and measurements for all cohorts were approved by the local ethics committee (Ethik-Kommission Ärztekammer Hamburg, approval numbers PV7142) and complied with the ethical standards of the Declaration of Helsinki.

### Statistics and study design

For the calculation of minimum sample size, the main outcome parameters derived by radiological and histological analysis were used, yielding a minimum group number of N = 5 mice. In this context, given the usual test approaches and laboratory conditions as well as the current lack of prior information (lack of prior probability), realistic error sizes and case numbers were assumed. With the acceptance of type I and II errors of 0.05 and 0.2, respectively, group sizes were determined using non-parametric test methods that allow the evidence of effect sizes between 1.5 and 1.8 with a standard deviation of 20%. Outliers are determined via Grubbs' test with a P value > 0.05 and excluded for further analysis. Groups were assigned randomly, and researchers were blinded during sample processing and analyses. Statistical analyses were performed using GraphPad Prism version 9.1.1 (GraphPad Software Inc., La Jolla, USA). Unpaired two-tailed Student’s t-test, one-way ANOVA or two-way ANOVA followed by Dunnett’s post hoc post-hoc test was used as indicated. Differences were considered statistically significant at *P* < 0.05.

## Data Availability

All relevant data of this project is presented within the figures of this manuscript. The data that support the findings of this study are available from the corresponding authors, TS and JK, upon reasonable request.

## Abbreviations

Panx1: Pannexin 1
Panx3: Pannexin 3
WT: Wild-type
µCT: Micro-computed tomography
BV: Bone volume
TV: Tissue volume
BS: Bone surface
BV/TV: Bone volume per tissue volume
CtTh: Cortical thickness
ObN/BPm: Osteoblast number per bone perimeter
ObS/BS: Osteoblast surface per bone surface
OcN/BPm: Osteoclast number per bone perimeter
OcS/BS: Osteoclast surface per bone surface
BSA: Bovine serum albumin
RT-qPCR: Real-time quantitative polymerase chain reaction
cDNA: Complementary DNA
RNA-seq: RNA sequencing
FCS: Fetal calf serum
αMEM: Alpha minimum essential medium
ELISA: Enzyme-linked immunosorbent assay
*Gapdh*: Glyceraldehyde-3-phosphate dehydrogenase
*C1qtnf3 *: C1q/TNF-related protein 3
*Glut4*: Glucose transporter 4 (Slc2a4)
*Glut1*: Glucose transporter 1 (Slc2a1)
*Dmp1*: Dentin matrix acidic phosphoprotein 1
*Alpl*: Alkaline phosphatase
*Bglap*: Bone gamma-carboxyglutamate protein (osteocalcin)
*Sp7*: Osterix
*Nos2*: Nitric oxide synthase 2
*Tnfa*: Tumor necrosis factor alpha
*Il6*: Interleukin-6
*Il10*: Interleukin-10
*Tgfβ*: Transforming growth factor beta
Cd31: Cluster of differentiation 31 (PECAM-1)
Emcn: Endomucin
TRAP: Tartrate-resistant acid phosphatase
VOI: Volume of interest
PBS: Phosphate-buffered saline
RNA: Ribonucleic acid
DNA: Deoxyribonucleic acid
A.U.: Arbitrary units
SPF: Specific pathogen-free
ARRIVE: Animal Research: Reporting of In Vivo Experiments
BMI: Body mass index
ANOVA: Analysis of variance
DFG: Deutsche Forschungsgemeinschaft
